# Adaptive characteristics of the gut microbiota of the scaly-sided merganser (*Mergus squamatus*) in energy compensation at different developmental stages

**DOI:** 10.3389/fmicb.2025.1614319

**Published:** 2025-07-30

**Authors:** Yanze Yu, Jiaming Wang, Luyi Shi, Hongyu Sun, Boxing Cheng, Yue Sun

**Affiliations:** ^1^School of Biological Sciences, Guizhou Education University, Guiyang, Guizhou, China; ^2^Wildlife Institute of Heilongjiang Province, Harbin, Heilongjiang, China

**Keywords:** waterfowl, gut microbiota, *Mergus squamatus*, metagenome, adult

## Abstract

The gut microbiota is crucial for maintaining health, enhancing digestive efficiency, and promoting the development of the immune system of the host. However, for the endangered waterfowl, the scaly-sided merganser (*Mergus squamatus*), the physiological role of the composition and structure of its gut microbiota during its growth and development remains unclear. Herein, we conducted fecal metagenomic analyses on adult and subadult populations to assess differences in the gut microbiota composition and function within the same habitat. The results revealed that this species harbors a diverse gut microbiota assemblage, with Firmicutes, Actinobacteria, Proteobacteria, and Bacteroidetes being the dominant phyla in adults and subadults. Notably, the abundance of the Firmicutes phylum is higher in adult, while the Actinobacteria phylum is more abundant in subadult individuals. There are significant differences in the diversity of the gut microbiota between the two age groups of the scaly-sided merganser. The alpha diversity index shows that the species richness and evenness of gut microbiota in adult scaly-sided merganser are higher than those in subadult individuals. Functional gene enrichment analysis further indicated that the adult gut microbiota had a higher ability to synthesize acetyl-CoA and pyruvate, along with enhanced conversion of acetyl-CoA to acetate. These findings suggest that the gut microbiota of the scaly-sided merganser can play a crucial role in concert with the host during the energy metabolism process in the growth and development stage. This study provides foundational data on the gut microbiota structure and function of this species and enhances our understanding of microbial dynamics during waterfowl development.

## Introduction

The gut of wildlife harbors complex microbial communities, whose interactions with the host are integral to nutrient absorption (Levin et al., 2021), growth and development (Zhang et al., 2025), immune defense (Lan et al., 2025), and behavioral regulation (Davidson et al., 2020). Given the critical role of gut microbiota on host biology, such as homeostasis maintenance and ecological adaptation, investigating its ecological and evolutionary implications is imperative (Ezenwa et al., 2012). The gut microbiota plays a crucial role in the host's energy metabolism, with one of the core mechanisms being the biosynthesis of short-chain fatty acids (SCFAs; Sanna et al., 2019). SCFAs mainly include acetate, propionate, and butyrate, which are the final metabolic products of the fermentation of dietary fibers and other complex carbohydrates by the gut microbiota and have a profound impact on the host's energy metabolism, immune regulation, and intestinal health (Cong et al., 2022; Louis and Flint, 2017). The generation of SCFAs is the result of complex interactions among dietary components, the composition of the gut microbiota, and the intestinal microenvironment (Dalile et al., 2019). Specifically, butyrate among SCFAs serves as the primary energy source for colonic epithelial cells (Brosolo et al., 2024).

The gut microbiota of birds, a highly successful evolutionary group with remarkable species and genetic diversity (Chen et al., 2020), are considered more unstable and adaptable than mammals (Hird, 2017). However, the integration of gut microbiota research into avian conservation biology remains limited (Mohsin Bukhari et al., 2022).

Previous research has shown that the dynamic modulation of gut microbiota is most likely the key mechanism for maintaining physiological balance and enhancing ecological adaptability in birds (Bodawatta et al., 2022). The relative abundance of specific gut microbes fluctuates with age and temporal factors in wild birds (Zhou et al., 2020; Spergser et al., 2018). Similarly, studies suggest that gut microbiota diversity during host development may follow non-linear trajectories, stabilize, or undergo marked fluctuations (Dewar et al., 2017). For example, in the critically endangered kakapo (*Strigops habroptilus*), amplicon pyrosequencing revealed higher abundances of lactic acid bacteria in the fecal microbiota of juveniles than in the adults, though the overall microcommunity structure remained consistent across age groups (Waite et al., 2014). Furthermore, the gut microbiota of wildlife undergo significant age-dependent shifts linked to phylogenetic traits and developmental stages, with pronounced compositional and functional differences between adults and subadults (Wang et al., 2019a,b). However, the role of age in shaping the avian gut microbiota remains understudied (Kashtanova et al., 2016). Research on gut microbiota in subadult wild bird populations is scarce due to challenges in sampling. To date, such studies have been limited to taxa such as seabirds (Barbosa et al., 2016), shorebirds (Grond et al., 2017), and passerines (Kohl et al., 2018). Notably, the gut microbiota dynamics between juvenile and adult waterfowls have not been studied yet. Therefore, a consensus on subadult and adult gut microbiota divergence is lacking, underscoring the need for further research on the developmental dynamics of the gut microbiota across avian taxa.

The scaly-sided merganser (*Mergus squamatus*), endemic to East Asia, inhabits mountainous regions below an elevation of 900 m (BirdLife International, 2017). This species breeds along clear flowing rivers within temperate coniferous-broadleaf forests, mainly relying on primary forests with abundant tree cavities for nesting (Zeng et al., 2018). In 2016, this species was assessed as “endangered” in the International Union for Conservation of Nature Red List of Threatened Species (Zeng et al., 2018). This species has been included in the “List of National Key Protected Wild Animals of China” and is classified as a first-class protected animal. However, the adaptive changes in the gut microbiota composition and function during different developmental stages of this species remain unclear.

In this study, we conducted metagenomic analyses on fresh fecal samples collected from the scaly-sided mergansers in the Bishui National Nature Reserve in Heilongjiang Province, China. These analyses aimed to (1) characterize the gut microbiota community composition and investigate synergistic differences in taxonomic profiles and energy metabolism-related functional genes between adult and subadult populations, and (2) understand the role of gut microbiota in maintaining physiological homeostasis across developmental stages to predict the growth-related phylogenetic adaptations. Elucidating the relationship between host age and gut microbiota dynamics is crucial for forecasting population health and refining biodiversity management efforts, which can enhance conservation strategies for this endangered waterfowl.

## Materials and methods

### Fecal sample collection

**Figure 1:**
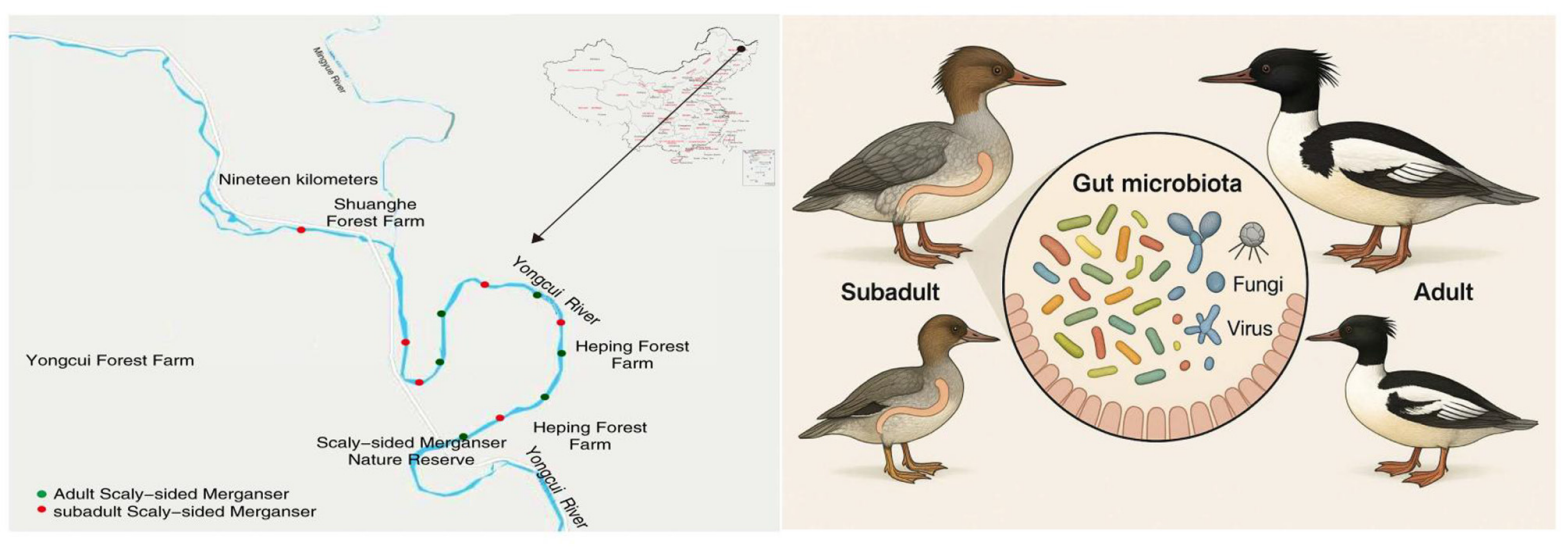
Schematic diagram of the research on gut microbiota of scaly-sided merganser at different age stages.

The fecal samples of scaly-sided merganser were collected from the Bishui National Nature Reserve (47.083611°-47.168611°N, 128.788056°-128.978889°E) in Heilongjiang Province, China. The Heilongjiang Bishui National Nature Reserve for scaly-sided merganser is located in the southeastern section of the Xiaoxing'anling Mountains in the northeastern part of China. As one of the most important breeding habitats for the scaly-sided merganser in China, the population in the reserve has remained stable at around 60–70 individuals in recent years, with ~25 pairs breeding each year (Yang et al., 2024). The population can reach around 100 during the southward migration in autumn. The subadult stage of the scaly-sided merganser usually refers to individuals from the time they leave the nest (about 60 days old) until they reach sexual maturity (typically 2–3 years old), and they have distinct physical features and coloration from adults (Yang et al., 2024). Studies have shown that Chinese mergansers exhibit significant differences in dietary preferences at different age stages (Yang et al., 2024). Subadults lack the prominent crest on the head observed in adult birds, and the scale patterns on their flanks are relatively indistinct. Male subadults display a less pronounced green luster on their heads, while female subadults are predominantly grayish-brown in coloration. Adult Scaly-sided Mergansers measure between 580 and 650 millimeters in total length, whereas subadults aged 1–2 years are ~90–95% of the size of adults (BirdLife International, 2017). In August 2023, we used telescopes to track and observe the behaviors of individual scaly-sided mergansers from a distance of about 5 m for a long time. When we observed the scaly-sided merganser resting and defecating on the stones in the middle of the river, we could directly distinguish adults and subadults by their plumage and body size. As the scaly-sided merganser are gregarious, we collected samples simultaneously from different areas of Yongcui River in the Bishui National Nature Reserve. In the early morning, after observing different scaly-sided mergansers resting and defecating through telescopes, we collected fresh feces within 2 h. We selected feces from adult and subadult scaly-sided merganser from different populations for metagenomic testing to ensure they came from different individuals. When defecation was observed, fresh feces (with mucous coating) were immediately collected and placed into labeled, sterile bags. All samples were flash-frozen at −80°C until DNA extraction and categorized into two groups: adult group (CT group, *n* = 6) and subadult group (YCT group, *n* = 6; Figure 1).

### Fecal DNA extraction, detection, and library construction

Genomic DNA was extracted from the fecal samples of the wild birds using the cetyltrimethylammonium bromide (CTAB) method. The DNA concentration, integrity, and purity were assessed using an Agilent 2100 Bioanalyzer (Agilent Technologies, USA). Sequencing libraries were constructed using the NEBNext^®^ Ultra™ DNA library prep kit for Illumina (New England Biolabs, USA; Catalog No. E7370L). The quality-checked DNA samples were sheared into 350-bp fragments using a Covaris S220 ultrasonicator (Covaris, USA), followed by end repair, A-tailing, adapter ligation, size selection, PCR amplification, and purification to complete library preparation. The PCR products were further purified with AMPure XP beads (Beckman Colter, USA), and library quality was verified using the Agilent 2100 system. The library concentration was quantified via real-time quantitative PCR (qPCR) with a sensitivity of 1.5 nM. Finally, the quantified libraries were sequenced on an Illumina NovaSeq 6000 platform with a paired-end 150 bp (PE150) strategy based on the optimal concentration and target data volume (Langmead and Salzberg, 2012).

### Data quality control

We performed metagenomic sequencing on the fecal samples of the waterfowl using the Illumina NovaSeq high-throughput sequencing platform. Raw metagenomic reads encompassing bacterial, fungal, and viral sequences were obtained and preprocessed with the Kneaddata software. The sequences shorter than 50 bp (parameter: MINLEN = 50) were filtered out. Data quality was validated using FastQC (Bolger et al., 2014).

### Species annotation

To determine the species composition in the samples, we analyzed the data using Kraken2 paired with a custom microbial database, which was constructed using the NCBI nucleotide and RefSeq whole-genome databases. Following rigorous filtering, sequences representing bacteria, fungi, archaea, and viruses were selected (Wood and Salzberg, 2014; Mandal et al., 2015; Brum et al., 2015). Species-specific relative abundance was quantified using Bracken.

### Common functional database annotations based on reads

We aligned the quality-controlled and decontaminated sequences against the UniRef90 protein database using HUMAnN2. After obtaining the annotation information, we generated relative abundance tables for each functional category using UniRef90 identifiers and their associations with multiple databases (Segata et al., 2011; Zhu et al., 2010; Kim et al., 2016).

### Statistical analysis

We quantitatively described the composition of each group of gut microbiota in the form of mean ± standard deviation (SD). We quantified microbial diversity using alpha diversity indices to assess species richness and coverage. The structural and functional variations in the gut microbiota of scaly-sided mergansers were assessed using PERMANOVA tests verify the significance of ecological distance matrices, the difference is visualized through Principal Component Analysis (PCA). Taxonomic and functional differences were identified through LEfSe analysis (*p* < 0.05, LDA > 4). Functional annotation of reads was conducted via the Kyoto Encyclopedia of Genes and Genomes (https://www.genome.jp/kegg/pathway.html) and the Human Metabolome Database (HMDB; https://hmdb.ca/metabolites; Xue et al., 2021), and hierarchical clustering of top-abundance features was visualized as a heatmap. The nitrogen cycle pathways were analyzed using the Wekemo Bioincloud platform. Differentially expressed metabolic pathways were detected via enrichment analysis (clusterProfiler package; fold change > 2, *p* < 0.05) and validated with STAMP software (Welch's *t*-test, *p* < 0.05; Villar et al., 2015). All analyses, including taxonomic profiling, alpha/beta-diversity assessments, and visualization, were conducted in R v4.1.2.

## Results

### The gut microbiota composition of scaly-sided mergansers

**Figure 2:**
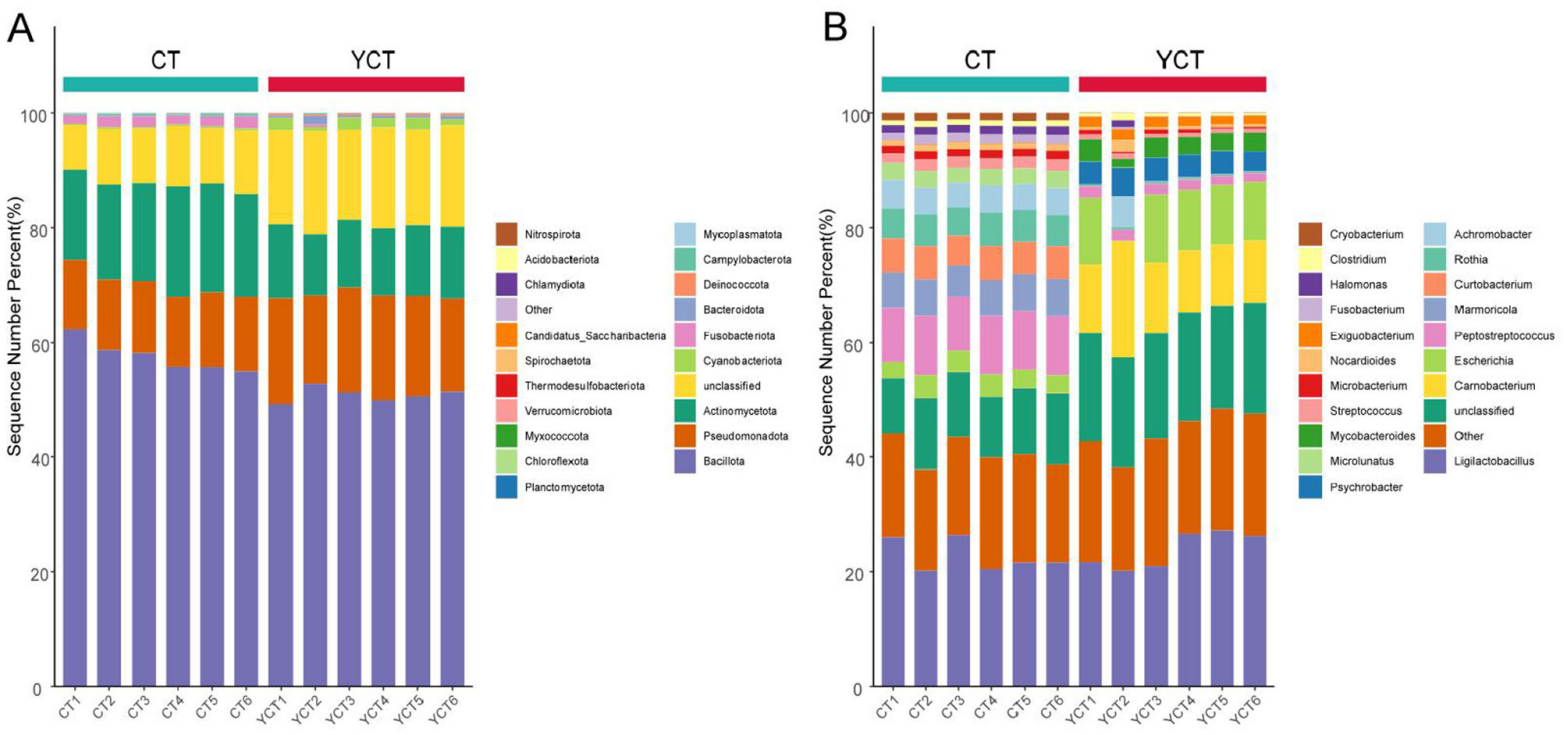
The structure composition of the gut microbiota of different age stages of the scaly-sided merganser. **(A)** Relative abundance of gut microbiota at the phylum level. **(B)** Relative abundance of gut microbiota at the genus level. Adults (CT group, *n* = 6) and subadults (YCT group, *n* = 6).

We collected 12 scaly-sided merganser fecal samples and obtained a total of 356,720,614 raw reads after metagenomic sequencing. After quality control, 274,984,583 clean reads remained, accounting for 95% of the raw data. Based on taxonomic annotation using the predicted non-redundant gene data by Kraken2, 4,645 species were identified. Through analysis and statistics, the most of species detected in the samples were Bacteria (91.29%). At the phylum level, bacterial communities were predominantly composed of Firmicutes (mean ± SD: adults = 59.72 ± 2.21%, subadults = 51.10 ± 1.79%), Actinomycetota (adults = 16.39 ± 0.7%, subadults = 11.86 ± 1.13%), and Pseudomonadota (adults = 12.25 ± 0.17%, subadults = 17.32 ± 1.73%; Figure 2A). At the genus level, the dominant bacterial genera included *Ligilactobacillus* (adults = 25.84 ± 4.93%, subadults = 0.1958 ± 0.0071%), *Carnobacterium* (adults = 0.09 ± 0.01%, subadults = 13.98 ± 5.37%), *Escherichia* (adults = 3.66 ± 0.58%, subadults = 7.17 ± 6.14%), *Achromobacter* (adults = 4.76 ± 0.41%, subadults = 1.82 ± 3.07%), and *Marmoricola* (adults = 6.39 ± 0.35%, subadults = 0.09 ± 0.05%; Figure 2B).

### Gut microbiota differences between adult and subadult scaly-sided mergansers

**Figure 3:**
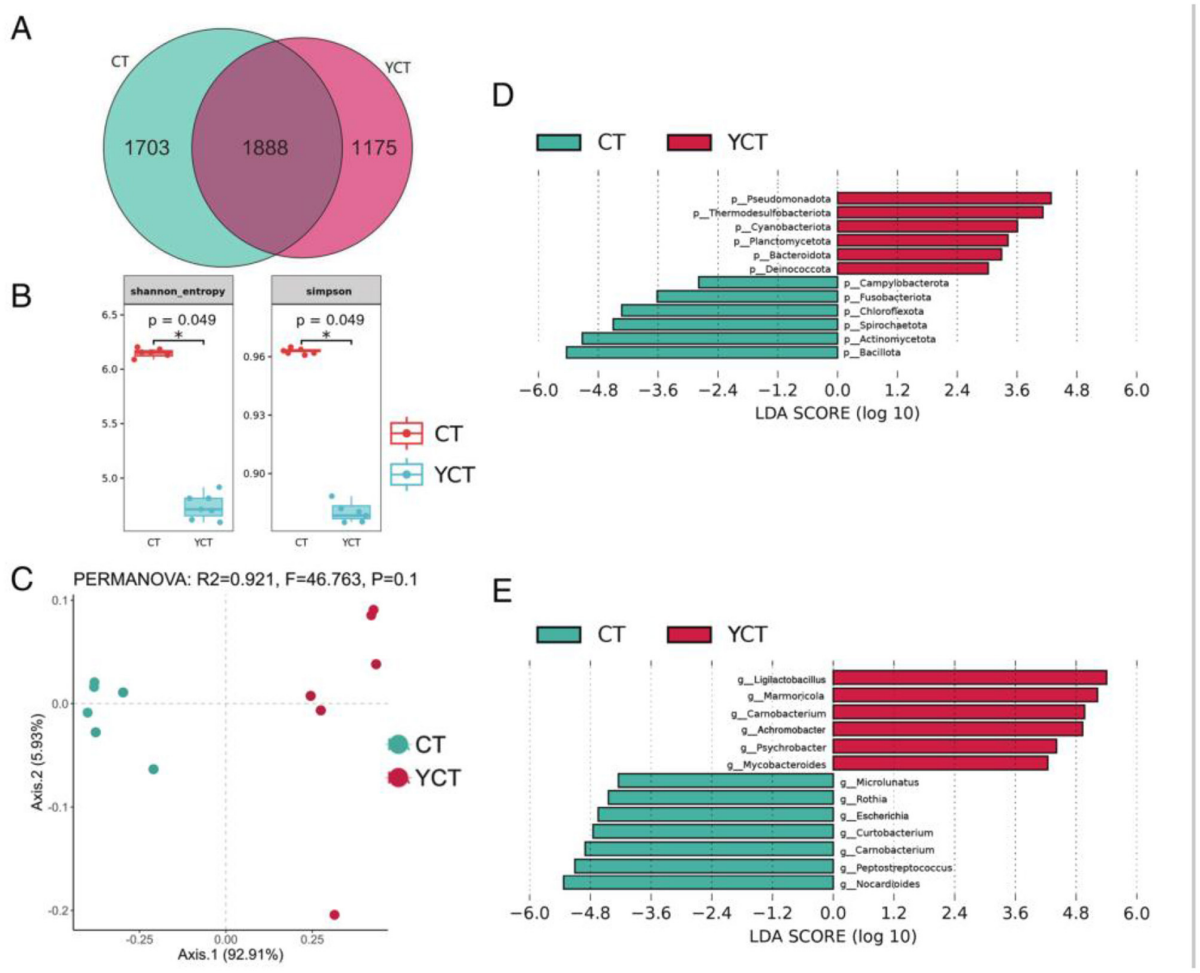
Differences in the composition and structure of gut microbiota among scaly-sided merganser of different ages. **(A)** Venn diagram showing unique and shared gut microbiota among the two groups of scaly-sided merganser. **(B)** Alpha diversity differences of scaly-sided merganser in different ages (Welch's *t*-test, *p* < 0.05). **(C)** Principal coordinate analysis (PCoA) of gut microbiota at the species level across ages (*p* < 0.05). **(D)** The LDA score distribution of phylum level differential relative abundance of gut microbiota (LDA > 4, *p* < 0.05). **(E)** The LDA score distribution of genus level differential relative abundance of gut microbiota (LDA > 4, *p* < 0.05). Adults (CT group, *n* = 6) and subadults (YCT group, *n* = 6).

The community structure of the scaly-sided merganser gut microbiota of the adult group differed significantly from that of the subadult group. The result revealed that 39.61% of the microbial communities were present in both the adult and subadult groups. This implied that the core microbiome exhibits high levels of abundance. According to the grouped data, 1,175 unique gut microbiota species of scaly-sided merganser were assigned to the subadult group, whereas 1,703 unique gut microbiota species were assigned to the adult group (Figure 3A). From the microbial community alpha diversity analysis, the results indicated that the gut microbiota at the species level showed significant differences between the adult and subadult groups (chao index, observed index, Shannon index, Simpson index; Welch's *t*-test, *p* < 0.05; Figure 3B). Thus, the gut microbiota richness and evenness of the adult group were higher than those in the subadult group. Furthermore, these dissimilarities in gut microbiota were further confirmed by principal coordinate analysis (PCoA) based on Bray–Curtis between the two groups (Figure 3C). The Linear Discriminant Analysis (LDA) Effect Size (LEfSe) analysis showed that there were significant differences in the abundance of phylum level of gut microbiota between the two groups (*p* < 0.05, LDA > 4; Figure 3D). Further, the LDA analysis of species level was found that the abundance of *Ligilactobacillus, Carnobacterium, Mycobacteroides*, and *Psychrobacter* was significantly higher in genus level of the subadult group. Specifically, the abundance of *Microlunatus, Aspergillus, Rothia, Curtobacterium, Marmoricola, Peptostreptococcus*, and *Nocardioides* was significantly higher in genus level of the adult group (*p* < 0.05, LDA > 4; Figure 3E). These results suggest that the gut microbiota was similar in composition at the core flora but its composition structure and diversity varied with the different developmental stages.

### Differences in gut microbiota functional genes between adult and subadult populations

**Figure 4:**
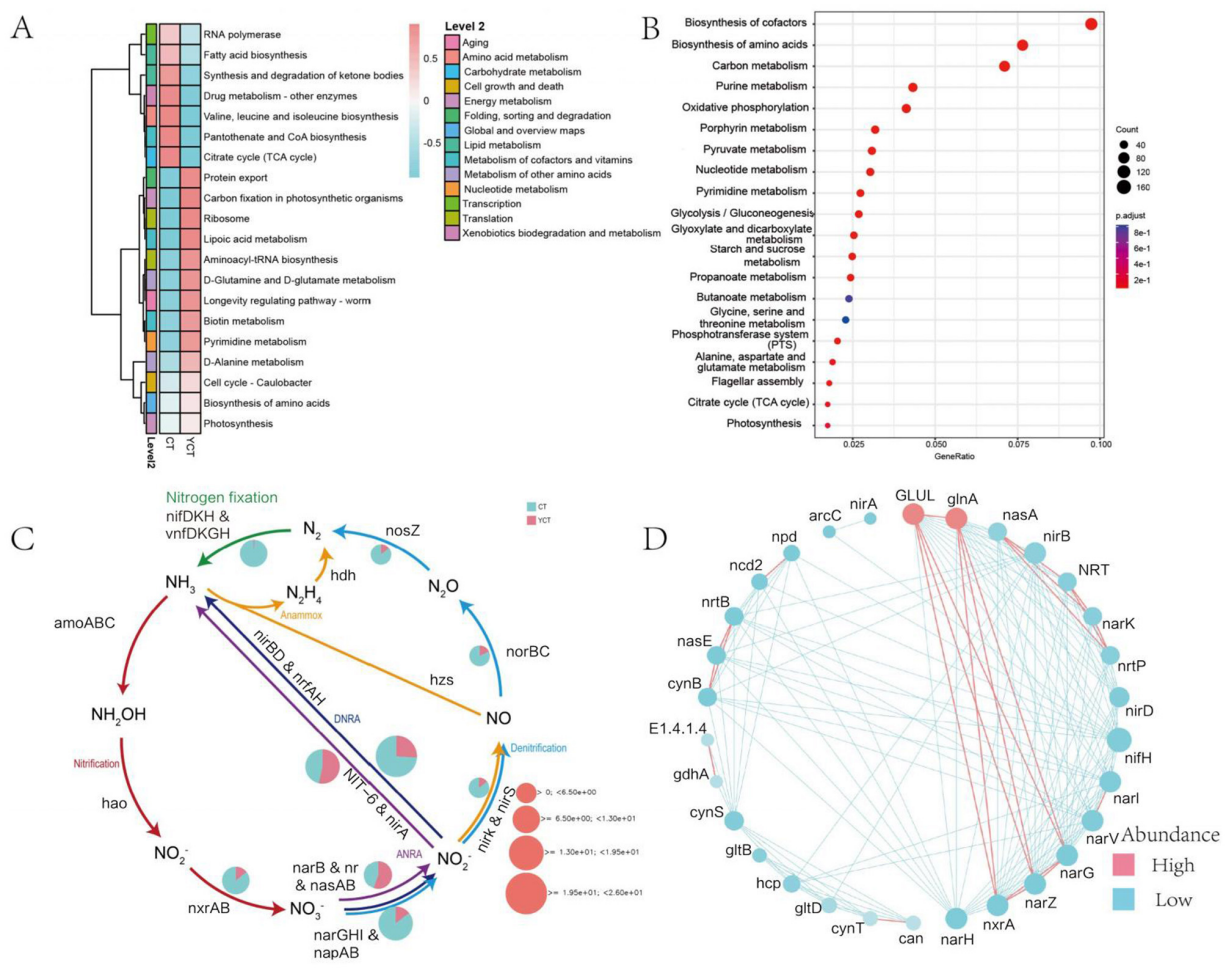
Functional gene composition of the gut microbiota in scaly-sided merganser. **(A)** Heat map of functional gene abundance levels of gut microbiota in scaly-sided merganser based on KEGG level 3 for two groups. **(B)** Bubble chart of enrichment analysis of functional genes (Log2 Fold Change > 1, *p* < 0.05). The bubble chart is colored based on the corrected *P* value (p.adjust value). The smaller the *P* value, the more significant the enrichment of the pathway. The color in the chart gradually changes from blue to red, indicating a decreasing *P* value and an increasing significance of enrichment. Additionally, the size of the bubbles reflects the Count value, which represents the number of differentially expressed genes in the pathway. The larger the bubble, the more differentially expressed genes are contained in the pathway. **(C)** Relative abundances of the pathways involved in the nitrogen cycle. The pie chart indicates the relative abundance of each pathway in each metagenomic sample. The size of pie charts represent the total relative abundance of each pathway. ANRA, asimilatory nitrate reduction to ammonium; DNRA, Dissimilatory nitrate reduction to ammonium; Anammox, anaerobic ammonium oxidation. **(D)** Network diagram of functional genes, where node size indicates importance, red represents a positive correlation, and blue represents a negative correlation. Adults (CT group, *n* = 6) and subadults (YCT group, *n* = 6).

Metagenomic sequencing delineated the scaly-sided merganser gut microbiota functional potential through UniRef90-based annotation, revealing 5,171 KEGG Orthology entries that were mapped to core metabolic pathways. Among the six functional categories in the KEGG level 1 pathway, the proportion of the metabolic category is the highest (61.68%), followed by genetic information processing (21.02%), cellular processes (5.39%), human diseases (5.45%), organismal systems (3.43%), and environmental information processing (2.53%; Supplementary Figure S1). Functional genes were annotated as being involved in 48 KEGG level 2 pathways, among which the top four in abundance were Translation, Amino acid metabolism, Carbohydrate metabolism and Metabolism of cofactors and vitamins (Supplementary Figure S2). Further, the heatmap revealed distinct functional clustering between adult and subadult groups (Supplementary Figure S3; Figure 4A). Key metabolic pathways including Valine, leucine, and isoleucine biosynthesis (log2 fold change = 3.2, subadult > adult) and Drug metabolism-other enzymes pathways (log2 fold change = 2.8, subadult > adult) were significantly enriched in the subadult group. In contrast, adult group showed higher abundance of environmental adaptation pathways like Photosynthesis pathway (log2 fold change = 4.1, adult > subadult). This indicates that scaly-sided merganser of different ages may achieve different functions of their gut microbiota by regulating different specific metabolic pathways. The enrichment analysis of functional genes revealed that in the three level metabolic pathways of the KEGG database, functional genes was significantly enriched in Biosynthesis of cofactors, Biosynthesis of amino acids, Carbon metabolism, Purine metabolism, Oxidative phosphorylation, Porphyrin metabolism, Pyruvate metabolism, and Nucleotide metabolism (Figure 4B; Supplementary Figures S4, S5; Log2FoldChange > 1, *p* < 0.05).

In addition, given the crucial role of nitrogen in energy metabolism, this study utilized the DiTing software to annotate the relative abundance of functional genes in the nitrogen cycling pathways. The results indicated that the relative abundance of the adult group in various nitrogen cycling pathways (such as nitrification, denitrification, etc.) was significantly higher than that of the subadult group (Figure 4C). The network analysis of functional genes shows that the GLUL, glnA genes have a relatively high abundance and play a significant role in the functional network, and at the same time, they are positively correlated with the nxrA, narZ, and narG genes (Figure 4D). These findings suggested that the abundance of different functional classes was significantly different between the adult and subadult groups, indicating that the gut microbiota of different age groups perform different functions.

### Differences in synthesis of energy substrates between adult and subadult populations

**Figure 5:**
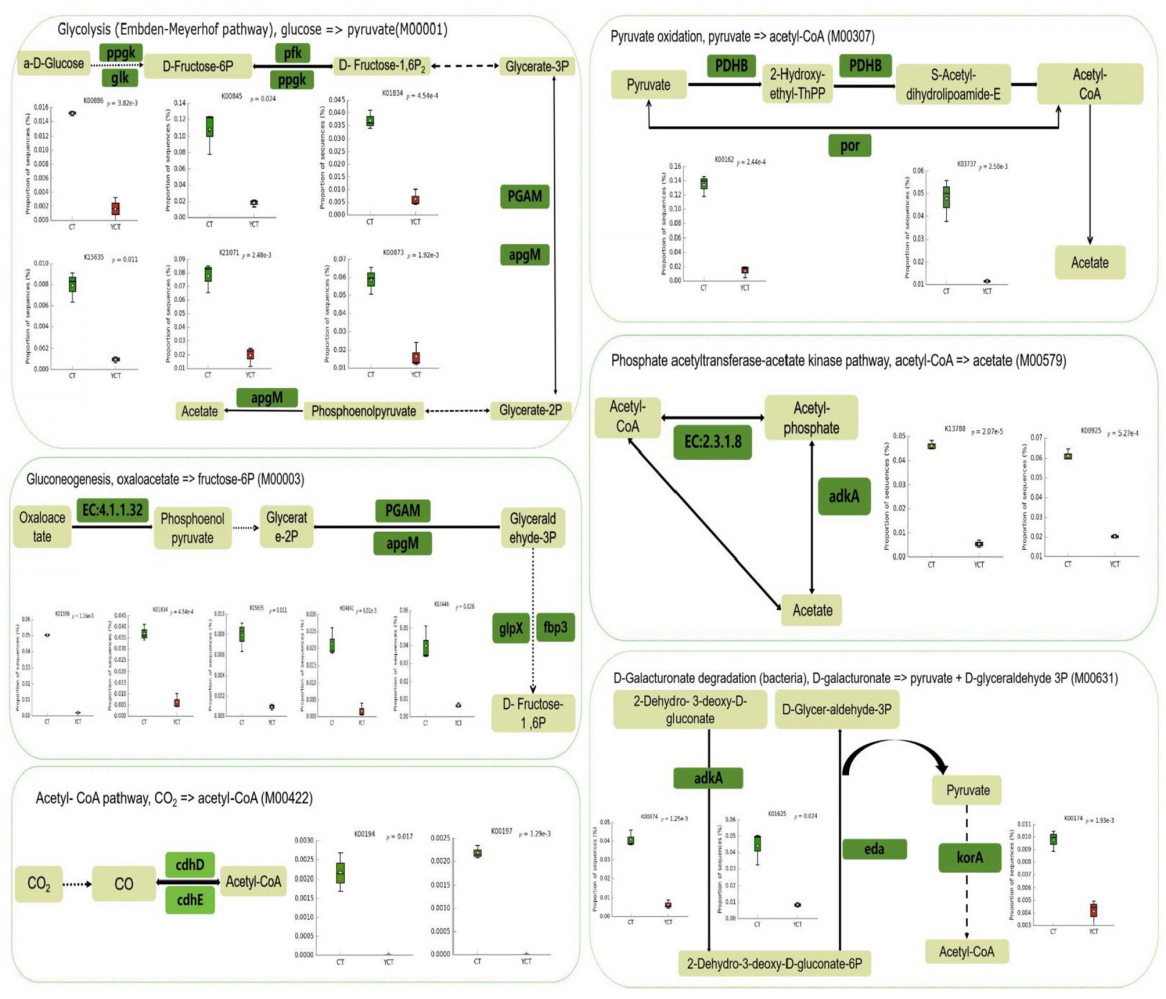
Schematic diagram of the main modules involved in differential gene expression. Green indicates a significantly higher relative abundance in adult, while red indicates a significantly higher abundance in subadult. Green Boxes represent the functional genes with significant differences (*p* < 0.05). The box plot represents the differentially expressed genes between adult and subadult individuals. It's represent 25th−75th percentiles, black lines indicate the median and whiskers extend to the maximum and minimum values within 1.5 × the interquartile range.

Furthermore, through the statistical analysis of metagenomic profiles (STAMP) for differential analysis, we found that the abundance of 1,619 KO genes was significantly different between the two groups (Welch's *t*-test, *p* < 0.05). The Color tool in KEGG Mapper was used for comparative analysis of the 1,619 KO genes. A total of 164 KOs associated with the “microbial metabolism in diverse environments (map01120)” pathway were identified. Of these, 127 functional genes exhibited higher abundance in the adult group, whereas 37 functional genes showed greater prevalence in the subadult group. At the same time, the 164 KOs were further screened and found to be involved in CO_2_ to acetyl-CoA (M00422), D-galactonate to pyruvate and D-glyceraldehyde 3P (M00631), pyruvate to acetyl-CoA (M00307) and oxaloacetate to fructose-6P (M00003) modules, which were significantly enriched in the adult group (Figure 5). Furthermore, the acetyl-CoA to acetate pathway (M00579), as well as butyrate synthesis-related ketone body biosynthesis module (M00088) extended pathway genes, were also significantly enriched in the adult group (*p* < 0.05). These results suggest that the gut microbiota in the adult group has a significantly higher potential to synthesize acetate and butyrate (Figure 5; Supplementary Figure S6).

In addition, we also found that the adult group was significantly enriched in genes related to Glycolysis (Embden-Meyerhof pathway), glucose = > pyruvate (M00001), Citrate cycle (TCA cycle, Krebs cycle; M00009), and Leucine degradation, leucine = > acetoacetate + acetyl-CoA (M00036; Figure 5; Supplementary Figure S6). The subadult group was also significantly enriched in genes related to Purine degradation, xanthine = > urea (M00546), and Guanine ribonucleotide degradation, GMP = > Urate (M00959; Supplementary Figure S6). These findings suggested that the gut microbiota of the scaly-sided merganser may work synergistically with the host to support energy compensation.

## Discussion

This study characterized the composition and functional profiles of the gut microbiota of scaly-sided mergansers across different developmental stages, comparing adult and subadult populations within the same habitat. Age-dependent variations emerged in microbial diversity, taxonomic abundance, and metabolic functions. Adults exhibited significantly richer alpha diversity and greater representation of the biosynthesis and energy metabolism pathways compared to the subadults.

Our findings revealed that the dominant phyla among the gut microbiota of the scaly-sided merganser comprised Firmicutes, Proteobacteria, Actinobacteria, and Bacteroidetes, consistent with those observed in other waterfowl species within Anatidae, including the mallard (*Anas platyrhynchos*; He et al., 2023), bar-headed goose (*Anser indicus*; Dong et al., 2022), Baer's Pochard (*Aythya baeri*; Xi et al., 2021), and whooper swan (*Cygnus cygnus*; Wang et al., 2021). The relative abundance of Firmicutes and Actinobacteria in adult scaly-sided mergansers is higher than that in subadults, which may be related to the more diverse diet of adult scaly-sided mergansers. Firmicutes have the ability to degrade complex polysaccharides (such as chitin), and this characteristic may be closely related to the energy extraction ability of adult individuals (Ley et al., 2008). In addition, Actinobacteria, due to their anti-inflammatory properties, may play an important role in maintaining the immune homeostasis of adult scaly-sided mergansers (Turroni et al., 2014; Sadeghi et al., 2024).

At the genus level, the gut microbiota of the scaly-sided mergansers exhibited significant compositional diversity, predominantly consisting of *Lactobacillus*, followed by *Peptostreptococcus, Escherichia, Achromobacter*, and *Marmoricola*. The abundance of *Lactobacillus* in adults was significantly higher than that in subadults, which might be closely related to its ability to produce various digestive enzymes (such as lipase and protease; Zheng et al., 2018). These enzymes help adults more efficiently break down and absorb nutrients. In contrast, the diet of subadults is more diverse, and their digestive systems are not yet fully developed, resulting in an unstable environment for *Lactobacillus* colonization. Previous studies have shown that the diversity of *Lactobacillus* strains in wild waterfowl increases with age (Zhang et al., 2020), and this cumulative effect might be the main reason for the higher abundance of this genus in adults. However, the relative abundance of *Carnobacterium* was higher in subadults, which might be attributed to *Carnobacterium*'s support for the rapid growth and development of subadults and its special adaptability to the metabolism of proteins and fats (Wang et al., 2016). Additionally, the abundance of *Marmoricola* was significantly higher in adults than in subadults, which might be related to the relatively stable diet of adults and the presence of organic compounds and nitrates in their food that *Marmoricola* can utilize, thereby promoting its proliferation in the adult gut (Lacombe-Harvey et al., 2018). These results suggest that the gut microbiota of the scaly-sided mergansers may be influenced by multiple factors, including environmental factors, dietary structure, and changes in age stages. Additionally, the exchange and transmission relationship between microorganisms in the water environment and gut microbiota may also have a significant impact on the composition and function of the gut microbiota.

In our study, alpha diversity analysis revealed a distinct divergence in the diversity and richness of the gut microbiota between the adults and subadults. This might be linked to the variations in the relative abundance of specific gut microbiota species across age groups (Zhou et al., 2020; Spergser et al., 2018). Previous studies across taxa have demonstrated that ontogenetic development substantially enhances the gut microbial diversity, indicating a conserved ecological pattern (Waite et al., 2014; van Dongen et al., 2013; Kohl et al., 2019). Furthermore, the high gut microbiota diversity seen in adult populations of wild pikas (*Ochotona curzoniae*) promotes the stability of the gut microbial ecosystem and enhances the digestion efficiency of the host (Tap et al., 2015; Li et al., 2019). Notably, even under identical environmental conditions, individuals of varying ages display significant differences in their core gut microbiomes, likely due to age-specific physiological or environmental factors influencing microbial composition (Gillingham et al., 2019; Wang et al., 2020).

Acetyl-CoA and pyruvate are core substances in cellular energy metabolism and play a crucial role in the metabolism of carbohydrates, fats, and proteins. Pyruvate, as the final product of glycolysis, is also an important precursor of acetyl-CoA; while acetyl-CoA is a key hub in energy metabolism, connecting the metabolic networks of the three major nutrients (Oliphant and Allen-Vercoe, 2019). Based on this, this study focuses on exploring the key energy metabolism modules related to the synthesis of acetyl-CoA and pyruvate.

In this study, we observed that the adult scaly-sided mergansers exhibit enhanced potential to synthesize energy substrates (specifically acetyl-CoA and pyruvate) via the metabolic modules M00422, M00631, M00003, M00009, and M00307. This suggests that the gut microbiota of these birds is crucial for modulating host energy metabolism and providing a compensatory energy supply. Comparative analysis of the energy metabolism-related functional genes associated with the gut microbiota between adults and subadults revealed that the adults demonstrated a higher capacity to synthesize acetyl-CoA from CO_2_ fixation (M00422 and M00009) and pyruvate (M00631, M00001, M00003, and M00307), and convert acetyl-CoA into acetate (M00579) and butyrate (M00088). This suggests that the gut microbiota of these adult birds is crucial for modulating host energy metabolism and providing a compensatory energy supply.

Combining the previous reports and our findings in this study, it is believed that some different external or internal factors have a huge impact on the difference in synthesizing energy substrates (specifically acetyl-CoA and pyruvate) between adults and subadults (Trivedi et al., 2015; Steeves and Gardner, 1999). As adult scaly-sided mergansers need to engage in high-energy-consuming behaviors such as courtship, nest-building, incubation and brood-rearing during the breeding period, they may have a higher demand for energy compensation, which is manifested as a higher potential for the synthesis of energy substrates than in other life stages (Elliott et al., 2014). Specifically, adult scaly-sided mergansers can directly synthesize pyruvate through glycolysis (M00001) and galacturonic acid degradation (M00631) pathways, thereby enhancing the potential for pyruvate production. Additionally, through the acetyl-CoA synthesis module (M00307 and M00422), the generation of acetyl-CoA can be further promoted. Glycolysis is the main source of pyruvate and also the starting point of energy metabolism. Under aerobic conditions, pyruvate is converted into acetyl-CoA (M00307) via the pyruvate dehydrogenase complex (Guo et al., 2024). As the starting point of the tricarboxylic acid cycle (M00009), acetyl-CoA is a core intermediate product in energy metabolism. After entering the tricarboxylic acid cycle, it can generate a large amount of ATP (Pettinato et al., 2022). Meanwhile, acetyl-CoA can also be reversely converted into pyruvate through gluconeogenesis (M00003), participating in the synthesis of host glucose, and can be transformed into acetic acid through the acetic acid synthesis module (M00579; Deja et al., 2024). Based on the key roles of the above-mentioned functional modules in energy metabolism, it can be inferred that the adult intestinal microbiota has a significant advantage in energy conversion. Specifically, the adult intestinal microbiota can more efficiently convert nutrients into energy, thereby better meeting the energy demands of adult daily activities and life-sustaining processes.

Based on the above analysis, the gut microbiota of adult scaly-sided mergansers exhibits a mature community structure, a stable microecological environment, and an efficient adaptability to diverse food sources, thereby demonstrating stronger substrate utilization and metabolic regulation capabilities (Yilmaz and Macpherson, 2025). In contrast, the gut tracts of subadults are still in the developmental stage, and their microbiota structure and function have not yet fully matured, which may to some extent limit the efficiency of acetyl-CoA synthesis. With age, the gut metabolic function gradually improves, and the ability to degrade and convert complex substrates such as dietary fiber significantly increases, thereby promoting the synthesis of energy substrates and short-chain fatty acids.

This study analyzed the potential functional differences of gut microbiota in the energy metabolism of scaly-sided mergansers at different age stages based on metagenomic data. However, this study has not yet clarified the direct action efficiency of gut microbiota on the host's energy metabolism through controlled experiments or other quantitative methods. Future research can combine technical means such as metabolomics and mass spectrometry analysis to directly detect the concentration changes of key metabolites in intestinal contents or plasma, so as to further reveal the specific mechanism of action and quantitative relationship between gut microbiota and the host's energy metabolism.

## Conclusion

In summary, this study reveals significant differences in the gut microbiota between adult and subadult scaly-sided mergansers. During the development from subadult to adult, the diversity of fecal microbial communities gradually increases, with the proportions of Firmicutes and Actinomycetota significantly rising, while the proportion of Pseudomonadota shows a decreasing trend. In adult individuals, we found that the ability of the gut microbiota to synthesize acetyl-CoA and pyruvate through M00001, M00003, M00009, M00422, and M00631 modules is enhanced, and the potential for energy metabolism to convert acetyl-CoA into acetate is significantly improved. These energy substrates may provide energy compensation for the host to meet higher energy demands in adulthood. This study not only demonstrates the adaptive evolution of scaly-sided mergansers in a specific ecological environment and their efficient energy utilization strategy but also reveals the synergistic mechanism of gut microbiota in meeting high energy demands during adulthood. Additionally, this study provides important basic data for exploring the structure and function of gut microbiota in wild waterfowl at different developmental stages and deepens our understanding of dynamic changes in gut microbiota during waterfowl development. A deeper understanding of physiological characteristics of this rare species will provide a scientific basis for future conservation efforts and health monitoring.

## Data Availability

The original contributions presented in the study are publicly available. This data can be found here: https://www.ncbi.nlm.nih.gov, accession number PRJNA1288570.
