# Supplementary material for: Adaptive characteristics of the gut microbiota of the scaly-sided merganser (Mergus squamatus) in energy compensation at different developmental stages
Source: Front Microbiol. 2025 Jul 30;16:1614319. doi: 10.3389/fmicb.2025.1614319 (PMC12344420; doi:10.3389/fmicb.2025.1614319)
Supplement: Supplementary file 1 [file Supplementary_file_1.docx]

**Supplementary Materials for**

**Adaptive Characteristics of the Gut Microbiota of the Scaly-sided Merganser (*Mergus squamatus*) in Energy Compensation at Different Developmental Stages**

**Authors**

Yanze Yu ^1,2,†,^ Jiaming Wang ^2,†^ Luyi Shi^2^ Hongyu Sun ^2,*^ Boxing Cheng^1,*^ and Yue Sun^1,*^

**Affiliations**

1 School of Biological Sciences,Guizhou Education University,115 GaoxinRoad,WuDang District,Guiyang 550018,Guizhou,China.

2 Wildlife Institute of Heilongjiang Province, 134 Haping Road, Nangang District, Harbin 150081, Heilongjiang, China.

* Corresponding Author:

Dr. Yue Sun, School of Biological Sciences,Guizhou Education University,115 GaoxinRoad,WuDang District,Guiyang 550018,Guizhou,China.

Email: [sy1028sy@163.com](mailto:sunyue@ioz.ac.cn)

Dr. Boxing Cheng, School of Biological Sciences,Guizhou Education University,115 GaoxinRoad,WuDang District,Guiyang 550018,Guizhou,China.

Email: cbx@gznc.edu.cn

Hongyu Sun,Wildlife Institute of Heilongjiang Province, 134 Haping Road, Nangang District, Harbin 150081, Heilongjiang, China;

Email: shy712@163.com

† These authors contributed equally to this work.

**
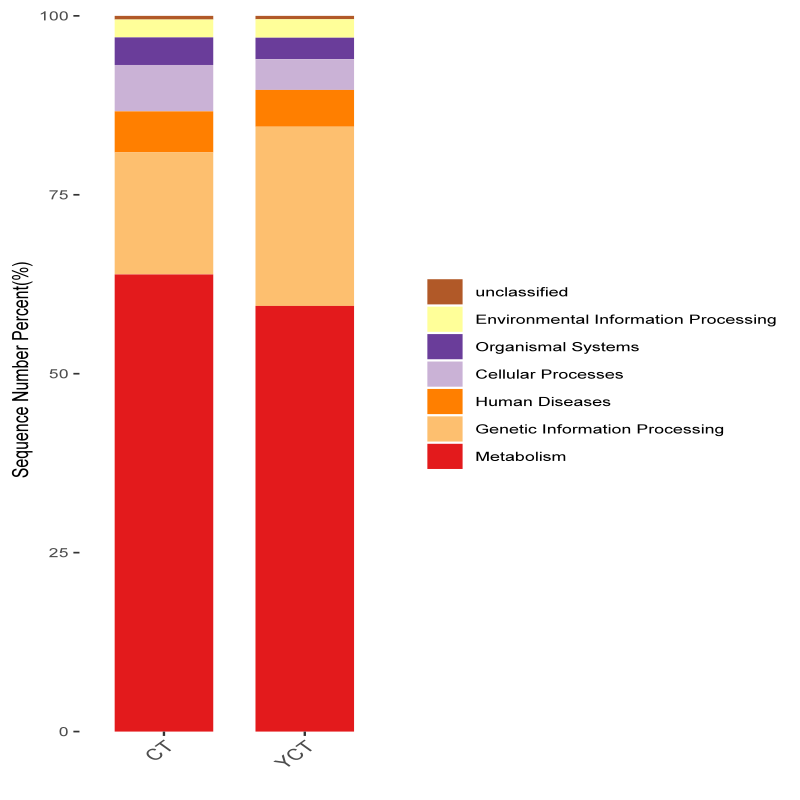
**

Supplementary Figure 1 | Distribution of KEGG Level 1 categories in the gut microbiota of Scaly-sided Mergansers by age group


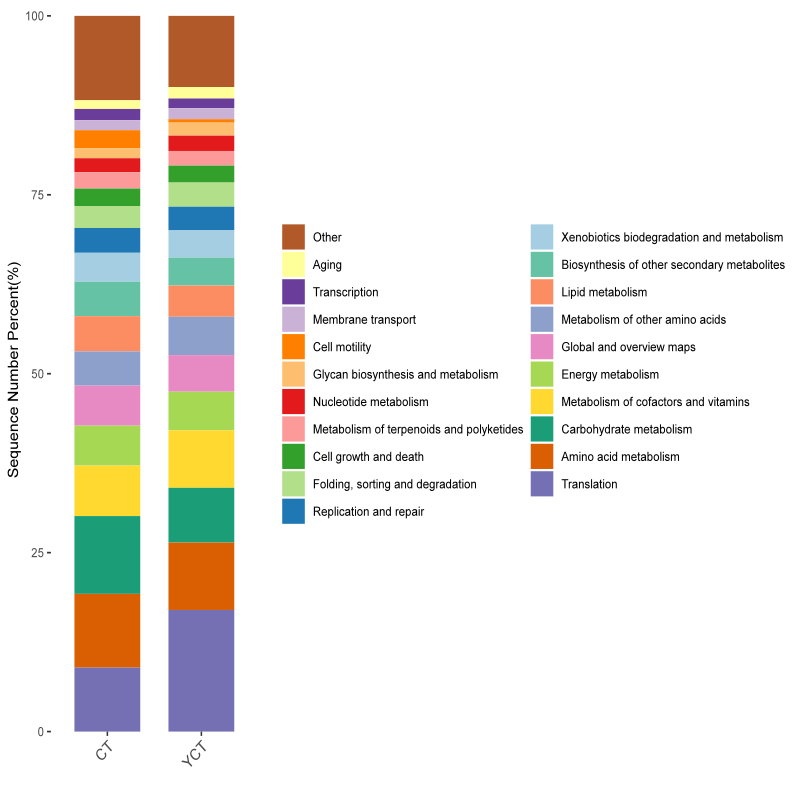


Supplementary Figure 2 | Distribution of the top 20 functional pathways in KEGG Level 2 categories in the gut microbiota of Scaly-sided Mergansers by age group


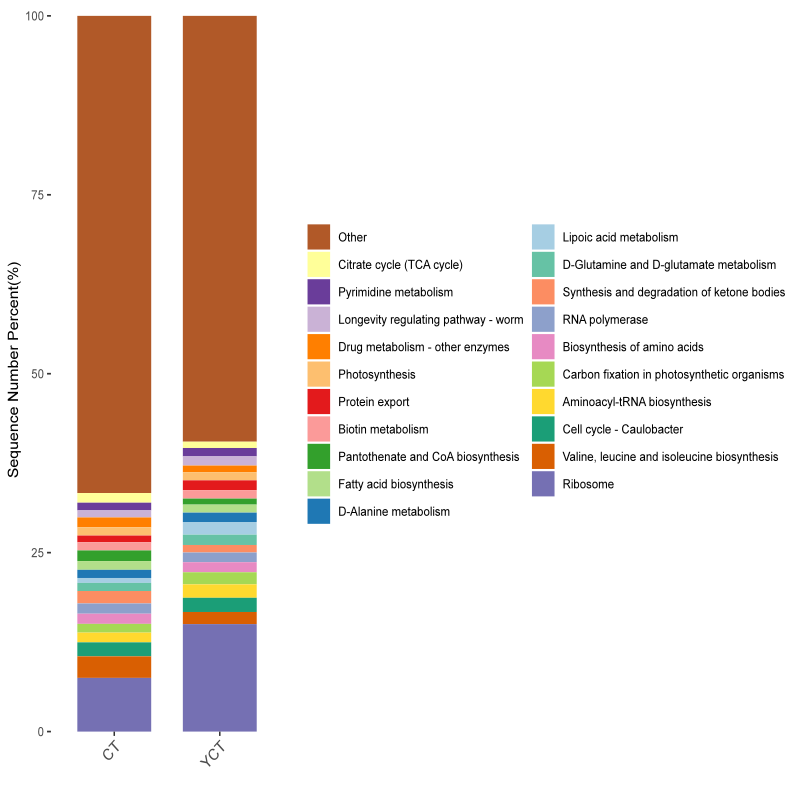


Supplementary Figure 3 | Distribution of the top 20 functional pathways in KEGG Level 3 Categories in the gut microbiota of Scaly-sided Mergansers by age group


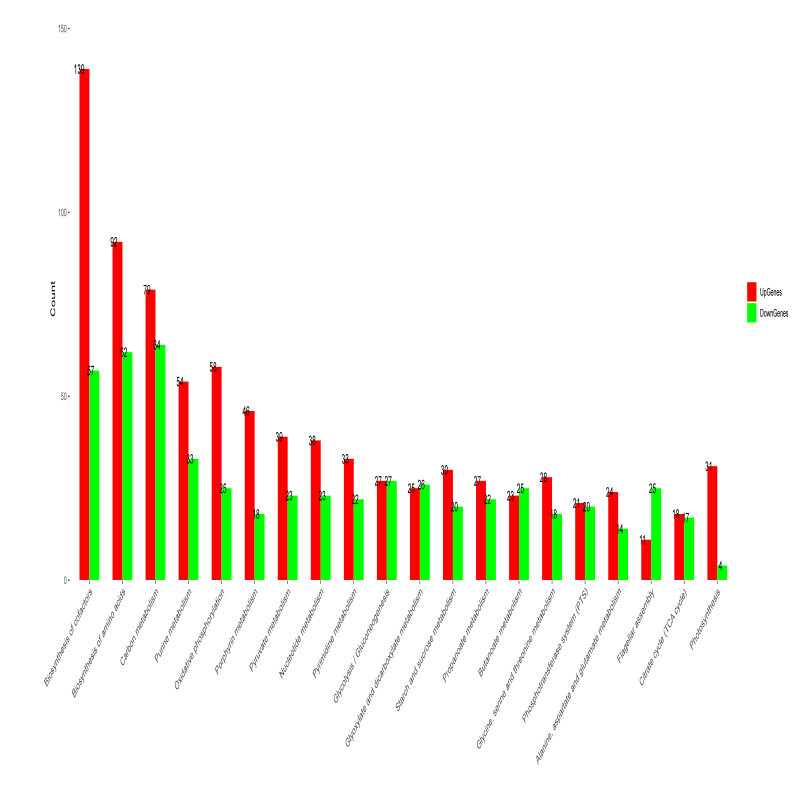


Supplementary Figure 4 | Functional gene enrichment analysis of the up-regulated expression of adult Scaly-sided Mergansersin energy metabolism pathways (Log2 FoldChange＞1, *p*＜0.05)


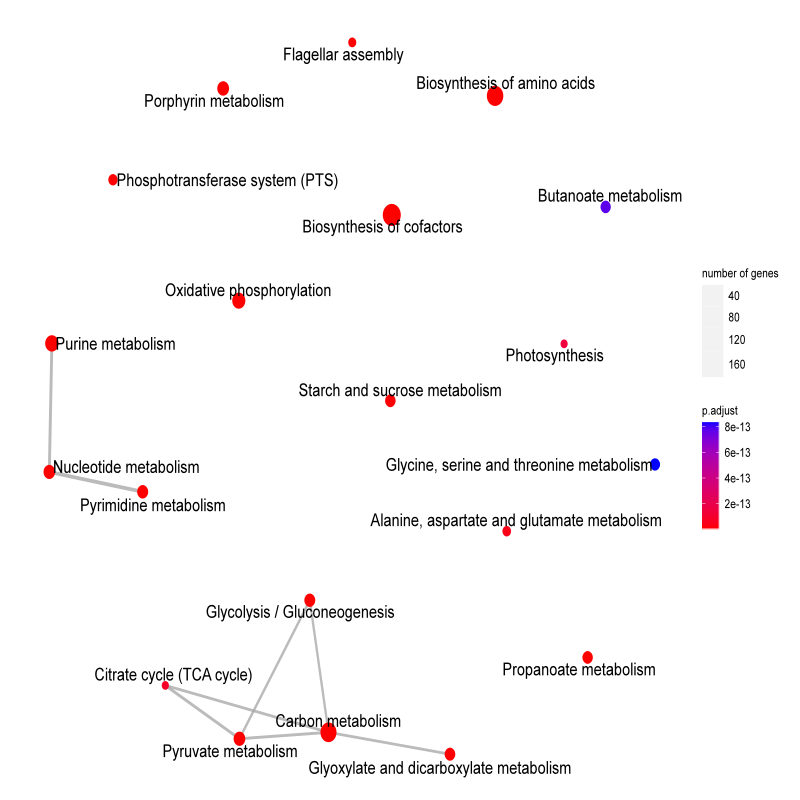


Supplementary Figure 5 | Functional gene enrichment analysis of the Scaly-sided Mergansersin energy metabolism pathways (Log2 FoldChange＞1, *p*＜0.05), The bubble chart is colored based on the corrected P value (p.adjust value). The smaller the P value, the more significant the enrichment of the pathway. The color in the chart gradually changes from blue to red, indicating a decreasing P value and an increasing significance of enrichment. Additionally, the size of the bubbles reflects the Count value, which represents the number of differentially expressed genes in the pathway. The larger the bubble, the more differentially expressed genes are contained in the pathway.


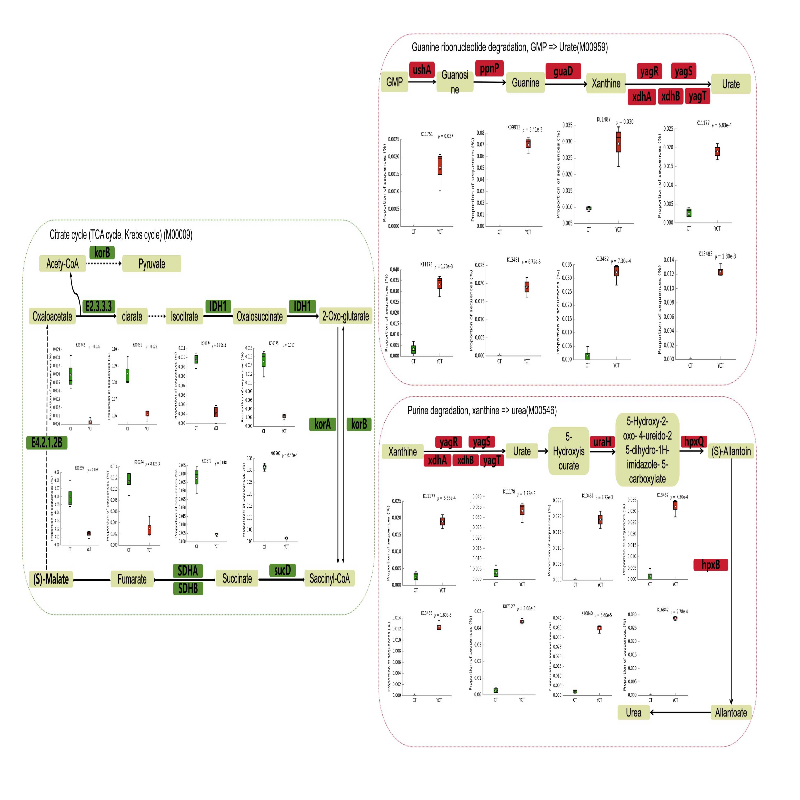


Supplementary Figure 6 | Schematic diagram of the main modules involved in differential gene expression Green indicates a significantly higher relative abundance in adult, while red indicates a significantly higher abundance in subadult. Green Boxes represent the functional genes with significant differences (*p* < 0.05). The box plot represents the differentially expressed genes between adult and sub-adult individuals. It’s represent 25th–75th percentiles,black lines indicate the median and whiskers extend to the maximum and minimum values within 1.5×the interquartile range

Supplementary Table 1 | The average abundance of functional genes in the nitrogen element cycling process

| Gene Family | CT group | YCT group | Description |
| --- | --- | --- | --- |
| K00360 | 0.940666667 | 0 | nasB; assimilatory nitrate reductase electron transfer subunit [EC:1.7.99.-] |
| K00362 | 23.261 | 6.292 | nirB; nitrite reductase (NADH) large subunit [EC:1.7.1.15] |
| K00363 | 15.19433333 | 5.956333333 | nirD; nitrite reductase (NADH) small subunit [EC:1.7.1.15] |
| K00366 | 8.823 | 9.051333333 | nirA; ferredoxin-nitrite reductase [EC:1.7.7.1] |
| K00367 | 0 | 5.0635 | narB; ferredoxin-nitrate reductase [EC:1.7.7.2] |
| K00368 | 2.2075 | 0.609666667 | nirK; nitrite reductase (NO-forming) [EC:1.7.2.1] |
| K00370 | 11.79333333 | 0.956333333 | narG, narZ, nxrA; nitrate reductase / nitrite oxidoreductase, alpha subunit [EC:1.7.5.1 1.7.99.-] |
| K00371 | 9.5355 | 2.525333333 | narH, narY, nxrB; nitrate reductase / nitrite oxidoreductase, beta subunit [EC:1.7.5.1 1.7.99.-] |
| K00372 | 8.641333333 | 0 | nasA; assimilatory nitrate reductase catalytic subunit [EC:1.7.99.-] |
| K00374 | 15.00766667 | 2.077666667 | narI, narV; nitrate reductase gamma subunit [EC:1.7.5.1 1.7.99.-] |
| K00376 | 4.0465 | 0.893 | nosZ; nitrous-oxide reductase [EC:1.7.2.4] |
| K02305 | 2.539666667 | 0 | norC; nitric oxide reductase subunit C |
| K02567 | 5.304 | 1.152 | napA; periplasmic nitrate reductase NapA [EC:1.7.99.-] |
| K02586 | 4.004 | 0 | nifD; nitrogenase molybdenum-iron protein alpha chain [EC:1.18.6.1] |
| K02588 | 18.532 | 0.298 | nifH; nitrogenase iron protein NifH |
| K02591 | 3.444 | 0 | nifK; nitrogenase molybdenum-iron protein beta chain [EC:1.18.6.1] |
| K03385 | 0 | 1.299 | nrfA; nitrite reductase (cytochrome c-552) [EC:1.7.2.2] |
| K04561 | 6.564 | 1.053 | norB; nitric oxide reductase subunit B [EC:1.7.2.5] |

Supplementary Table 2 | The average abundance of pathways in the nitrogen element cycling process

| Pathways | CT group | YCT group | Genes |
| --- | --- | --- | --- |
| Dissimilatory nitrate reduction, nitrate -> nitrite (narGHI or napAB) | 11.901 | 3.0595 | K00370,K00371,K00374,K02567,K02568 |
| Dissimilatory nitrate reduction, nitrite -> ammonia (nirBD or nrfAH) | 19.4915 | 7.96675 | K00362,K00363,K03385,K15876 |
| Assimilatory nitrate reduction, nitrate -> nitrite (narB or NR or nasAB) | 4.50675 | 5.0635 | K00367,K10534,K00372,K00360 |
| Assimilatory nitrate reduction, nitrite -> ammonia (NIT-6 or nirA) | 8.823 | 9.051333333 | K17877,K00366 |
| Denitrification, nitrite -> nitric oxide (nirK or nirS) | 2.2075 | 0.609666667 | K00368,K15864 |
| Denitrification, nitric oxide -> nitrous oxide (norBC) | 5.18675 | 0.4585 | K04561,K02305 |
| Denitrification, nitrous oxide -> nitrogen (nosZ) | 4.0465 | 0.893 | K00376 |
| Nitrogen fixation, nitrogen -> ammonia (nifKDH) | 8.654 | 0.099333333 | K02586,K02588,K02591,K22896,K22897,K22898,K22899 |
| Nitrification, nitrite -> nitrate (nxrAB) | 10.489 | 1.740833333 | K00370,K00371 |
